# Picturing the populations who could benefit from health insurance access expansions: An analysis of US health insurance television ads airing in 2018

**DOI:** 10.1002/wmh3.549

**Published:** 2022-09-12

**Authors:** Margaret Tait, Cynthia Pando, Cydney McGuire, Sarah Perez-Sanz, Laura Baum, Erika Fowler, Sarah Gollust

**Affiliations:** 1 University of Minnesota School of Public Health, Division of Health Policy and Management, Minneapolis, Minnesota, USA; 2 Indiana University Paul H. O’Neill School of Public and Environmental Affairs, Bloomington, Indiana, USA; 3 Berkeley Media Studies Group, Berkeley, California, USA; 4 Wesleyan Media Project, Middletown, Connecticut, USA; 5 Department of Government, Wesleyan University, Middletown, Connecticut, USA

**Keywords:** health reform, media, Medicaid

## Abstract

Efforts to expand access to health insurance in the United States are key to addressing health inequities and ensuring that all individuals have access to health care during the coronavirus disease 2019 pandemic. Yet, attempts to expand public insurance programs, including Medicaid, continue to face opposition in state and federal policymaking. Limited policy success raises questions about the health insurance information environment and the extent that available information signals both available resources and the need for policy reform. In this study, we explore one way that consumers and policymakers learn about health insurance—television advertisements—and analyze content in ads that could contribute to an understanding of who needs health insurance or who deserves to benefit from policies to expand insurance access. Specifically, we implement a content analysis of health insurance ads airing throughout 2018 on broadcast television or national cable, focusing on the depictions of people in those ads. Our findings indicate that individuals depicted in ads for Medicaid plans differ from those in ads for non-Medicaid plans. Groups that comprise large populations of current Medicaid enrollees, children and pregnant people, were more likely to appear in ads for non-Medicaid plans than in ads for Medicaid plans. This has implications for potential enrollees’ understanding of who is eligible as well as the general public’s and policymakers’ perspectives on who should be targeted for current or future policies.

## INTRODUCTION

Media—encompassing advertising, news, social media, and entertainment—play a complex role in shaping public understanding of health and social policy. Research reveals that media outlets provide cues to the public about interpreting and forming attitudes about policy (Jensen & Petersen, 2017; Rose & Baumgartner, 2013). Further, the media can initiate or reinforce beliefs about who deserves policy benefits (Brady & Sniderman, 1985; Gilens, 1999). In the context of health care policy in the United States, the media may depict populations in need of one specific type of policy benefit—access to health insurance—and the media may also shape the public’s attitudes about who deserves new or existing policies, such as Medicare for All or state-level expansions to the Medicaid program. If media depict policy beneficiaries (such as current or potential health insurance plan enrollees) as those commonly perceived by the public as deserving—such as the elderly, pregnant women, and children (A. L. Schneider & Ingram, 2005)—public opinion may reflect support for policy reform. Counter to this idea, if media show individuals and family structures viewed as undeserving of policy benefits—including people who are not working, single parents, or individuals with a history of criminal activity (Appelbaum, 2001)—public opinion may reflect less support for policy change.

In this study, we examine data from one high-volume media type: advertisements from public and private sponsors marketing health insurance plans, including Medicaid plans, aired in the United States in 2018. The objective is to describe the messages this media type explicitly and implicitly conveyed about who has and deserves health insurance in 2018, a time when US policy debates about health insurance and the Medicaid program were dynamic and contested. Medicaid is a federal entitlement program administered by states to provide health insurance coverage to eligible adults with low incomes, children, pregnant women, elderly adults, and individuals with disabilities (Centers for Medicare & Medicaid Services, 2021b). We seek to extend evidence of how the media socially construct target populations in the context of health care policy by exploring how health insurance advertisements depict Medicaid plan enrollees, and by extension, those who benefit from Medicaid. By examining the content of depictions of target populations receiving health insurance in a high-volume media type, this study contributes insights into the influences on public opinion of one element of ongoing health insurance reform efforts, such as expansions of state Medicaid programs.

## BACKGROUND

### The role of media in social constructions of target populations

Lippman (1922) posited that the mass media play a large role in shaping “the pictures in our heads” and our understanding of reality (van Doorn & Bos, 2017). Through print or visual depictions of individuals or groups in connection with content related to public policy, media also establish and reinforce ideas of who could or should benefit from policy. Media thus contribute to the social construction of target populations. Social constructions refer to the “…stereotypes about particular groups of people that have been created by politics, culture, socialization, history, the media, literature, religion, and the like” (A. Schneider & Ingram, 1993; p.335). In the context of the individuals or populations targeted by public policies, social constructions reflect “the cultural characterizations or popular images of the persons or groups whose behavior and well-being are affected by public policy” (A. Schneider & Ingram, 1993; p.334). It follows that the rhetoric surrounding and images of policy beneficiaries appearing in media related to health care policy could influence public opinion on the need for and possibility of policy reform.

Abundant previous work has examined how media depict target populations of social policy topics. For example, Gilens (1999), in his ground-breaking studies of poverty-related media, illuminated how the news media were more likely to depict Black individuals as poor and how this did not align with data about who was actually poor. Another study analyzed the photographs that accompanied stories on poverty in five US-based news magazines from 1993 to 1998. Similar to Gilens’ earlier work, the authors found that Black individuals were disproportionately portrayed as poor and largely overrepresented in stories that detailed the poor in stereotypical ways, such as stories on the cycle of dependency on welfare programs (Clawson & Trice, 2000). These and other studies offer evidence of the ways media misrepresent poverty and related social issues, and disproportionately focus on people of color. Such misrepresentations introduce and reinforce racist ideas that manifest in structural racism, which is “the totality of ways in which societies foster racial discrimination through mutually reinforcing systems of housing, education, employment, earnings, benefits, credit, media, health care, and criminal justice” (Bailey et al., 2017; p. 1453).

Research exploring the intersection of racialized and gendered media, including analysis of depictions of the stereotypical “welfare queen,” is a prominent example of how media systematically establish ideas of who benefits from public policy and how these ideas inform perceptions of who deserves to benefit (Gilens, 1999). Ronald Reagan popularized the term “welfare queen,” although it originally appeared in *The Chicago Tribune* in the 1970s as a way of describing suspected fraud in public assistance programs and perpetuating racist stereotypes (Gilman, 2014; Levin, 2013). The effects of racialized and gendered television news coverage about welfare on public attitudes about policy reform have been tested empirically, including with one experiment centered around the fictional story of a mother on welfare, Rhonda Germaine. In this study, participants viewed a portion of a fake newscast with Rhonda’s story. Some were shown a newscast which depicted Rhonda as a Black woman, which reinforced the “welfare queen” stereotype, whereas others were exposed to a newscast where Rhonda was depicted as a White woman or where no visual cue was provided. Among White participants, exposure to a Black Rhonda increased opposition to welfare spending by 5% and was associated with a 10% rise in an attribution of the cause of poverty to individual failings (Gilens, 1999). These findings suggest the power of media in initiating the images in our heads and how these images can contribute to false and racist ideas of poverty and its causes, as well as ideas of who should benefit from programs seeking to reduce poverty.

### Relevant research on health care policy-related media

The aforementioned research was not focused on health care policy beneficiaries. In the present study, we explore media content related to health care policy—specifically, the Medicaid program—and discuss the potential for content to influence ideas of who deserves access to health insurance. Below, we provide context on current US health care policy and politics, and a review of existing evidence on health care policy-relevant media.

The Patient Protection and Affordable Care Act (hereafter, the ACA) was passed in March 2010 during the Obama presidency and has been one of the most significant health care policy achievements of the twenty-first century. More than 20 million people gained access to health insurance coverage through the ACA (Center on Budget and Policy Priorities, 2019), many due to state-level expansions to the Medicaid program (Kaiser Family Foundation, 2020). The politics of health care, however, remain increasingly racialized and thus limit the potential for health outcomes proportionate to the significance of this policy achievement (Michener, 2021). Specifically, large segments of the public remain resistant to ideas of expanding access to health insurance (Kaiser Family Foundation, 2021) and scholars have provided evidence of how Americans’ racist attitudes affect public opinion of the need for health care policy reform (Mitchell & Dowe, 2019), perceiving reform as less of a priority when populations of color benefit (Knowles et al., 2010; McCabe, 2019). Republican policymakers at all levels of government have opposed expansions to the Medicaid program (Cassidy, 2017) and many have been part of efforts to propose restrictive and racist policies such as work requirements that limit eligibility, disproportionately burden individuals of color, and play to partisan fallacies about work and health (Alker et al., 2018; Grogan & Park, 2017; Haeder et al., 2021).

The media have both reflected and reified this dynamic political environment surrounding health care. Reviewing multiple media types (including local news and advertising), Gollust et al. (2019) synthesized 10 years of media messaging about the ACA and concluded that messaging has been complex and competitive, partisan, and of variable volume over time. In one previous study analyzing local news specifically, researchers provide evidence that coverage during the early years of ACA implementation was more likely to focus on political disagreement than on benefits of the law that might encourage enrollment, or on the populations who might benefit (Gollust et al., 2017, 2019).

Few studies have looked specifically at individuals depicted in ACA-related media content and what this suggests for public opinion of health care reform efforts to expand access to insurance. In one study, Viladrich (2019) analyzed *New York Times* articles published before and after the passage of the ACA, to understand how media frame access to health care for undocumented immigrants and suggest how these frames could influence perceptions of the need to include undocumented immigrants in future health care reform efforts. Analyzing content printed between 2009 and 2017, Viladrich concludes, “…as portrayed by the NYT stories reviewed in this article, undocumented immigrants must prove their deservingness for public health benefits on the basis of their moral worth, vulnerability, and need” (p.1455). This content could suggest that access to health insurance is a benefit that should be extended only to select populations, and conditionally so (Viladrich, 2019).

Looking specifically at the content of health insurance television advertisements aired during the first three open enrollment periods associated with the ACA (fall 2013 through spring 2016), Barry et al. (2018) examined who was depicted in ads, focusing on demographic depictions of up to five people per ad: female or male; race; life stage (e.g., child, young adult, older adult); disability; and body type, specifically whether they were overweight or obese (p.968). They found that Spanish ads were “…more likely (than English ads) to include non-White people, females, children and young adults, people exercising and people receiving medical care,” (p.7). Less frequent depictions of people of color and children in English-language ads could send the implicit signal that these are not the populations in need of health insurance, although recent data suggests otherwise. People from marginalized racial groups, including those who identify as Hispanic but may not speak Spanish (and as so, not receive information presented in Spanish-language ads) are at greater risk of being uninsured compared with their White non-Hispanic counterparts and more than a third (35%) of Hispanic children are uninsured (Tolbert & Orgera, 2020).

It is important to add that although many have gained coverage as a result of the ACA and expansions to state Medicaid programs, not all individuals who are eligible for program benefits enroll in Medicaid (Collins et al., 2016). Of the individuals who are eligible for Medicaid and remain uninsured, more than half (59.9%) are nonelderly adults and the remaining (40.1%) are children. Considering the uninsured and eligible nonelderly adults, the largest proportion are Hispanic (40%), followed by individuals who identify as White (35.2%), Black (15.8%), or respond with “Other” (9.0%) (Orgera et al., 2021). The reasons for limited enrollment are many: the administrative complexity of the program, of applying for, receiving, and maintaining benefits; perceptions of stigma associated with enrolling in a public program; as well as a lack of knowledge about available health insurance options and enrollment-related resources (Stuber & Bradley, 2005; Wright et al., 2017). In discussing the opportunities to increase enrollment among eligible populations, Wright and co-authors note, “These potential barriers suggest that targeted outreach that raises awareness …. might increase take-up,” (p.839). In their 2020 study, Shafer et al. (2020) find a relationship between health insurance advertising and health insurance enrollment, including a strong correlation between airings of private-sponsored ads mentioning Medicaid and enrollment in the Marketplace. This evidence, combined with other studies (e.g., Gollust et al., 2018; Karaca-Mandic et al., 2017) demonstrates the importance of health insurance advertising and enrollment, and motivates our study on the content of advertising. Ads about the Medicaid program may be one opportunity to increase awareness of the program among eligible populations, not only because of their direct messages urging enrollment (see, e.g., Pando et al., 2022) but also because of the images they provide about who is (and is not) an enrollee. In addition to providing information about the program and direct messages about enrollment (see, e.g., Pando et al., 2022), advertisements provide images about who is—and is not—an enrollee; accurate depictions of the populations enrolled in and eligible for coverage could contribute to bolstering program uptake.

### Study motivation and research questions

The current study contributes to previous health insurance policy-related media content analysis studies in a few key ways. First, we examine more recent advertising content (2018) during a time of political uncertainty around health insurance; specifically, this was a time of concerted administrative efforts to erode the impact of health insurance expansions, including through eliminating federal advertising completely (Kliff, 2017), and during a political campaign season characterized as the “health care election” following failed Congressional attempts to repeal and replace the ACA (Cillizza, 2018). Second, we compare advertisements specifically mentioning Medicaid to content that does not mention Medicaid. Existing studies have primarily analyzed media content when the ACA was relatively new—when the federal government (under the Obama administration) invested in health insurance television advertising and outreach in an effort to increase access to health insurance. As noted above, the federal government under the Trump Administration drastically reduced their investment in marketing for the 2018 open enrollment period (the period relevant to the analyses presented in this paper) (Gollust et al., 2018). With such reduced federal investment, most advertisements aired (and thus in this study) were instead sponsored by private insurance companies promoting their health plans (whether on the individual market or not). With regard to Medicaid plans in particular, a large proportion of private sponsors promoted Medicaid-managed care plans. These predominantly private entities were effectively shaping the “pictures” in the public’s heads—to borrow from Lippman (1922)—of who currently deserves access to health insurance through Medicaid and who could benefit as result of expansions to the ACA or other policies, all during a period when the federal government’s support for health insurance was particularly unpredictable.

Our first research question asks whether the individuals depicted in ads for Medicaid plans differ from those depicted in other ads’ content. Our second research question asks whether those depictions align with the populations currently enrolled in Medicaid.

## MATERIALS AND METHODS

### Data

This study used data from Kantar/Campaign Media Analysis Group (CMAG), which we obtained from the Wesleyan Media Project (https://mediaproject.wesleyan.edu/dataaccess/). Kantar/CMAG tracks 936 predominantly English-language television stations across all 210 designated market areas (DMAs) and 108 Spanish-language television stations across 38 DMAs in the United States (Pintor et al., 2020). Kantar/CMAG had data for 1723 health insurance-related advertisements that aired 877,318 times between January 1 and December 21, 2018, on broadcast television or national cable across all DMAs. Data from 2018 were the most recent complete data available to our team at the time of this analysis. In the text that follows, we refer to individual advertisements as a creative; this term is used by the data provider and within the advertising industry. We excluded Medicare-focused creatives as they were not relevant to our research questions. This left us with 960 advertisements that aired 489,489 times during this period, which is broader than but encompasses the 2019 Healthcare.gov open enrollment period, November 1–December 15, 2018.

Our sample was drawn to allow for maximum content variability. Although this analysis does not present differences in ad content by language spoken, our sample does contain all the Spanish-language creatives (*N* = 189) for this time period. To maximize the variability of content, as well as to make the best use of available resources, we developed and implemented the following sampling strategy. First, we drew a random sample of 46% of the total ads (English and Spanish language) furnished by the top three most common private sponsors. Ads sponsored by these insurers—Blue Cross/Blue Shield, United Healthcare, and University of Pittsburgh Medical Center—supplied more than a third (37%; *N* = 358 creatives) of the total unique ads in the data set. Our decision was justified, because we observed during the pilot coding stage that many of these ads were duplicative and included only subtle distinctions, which were not relevant to our research interests (e.g., referring to different locations plans were available). Creatives sponsored by the three largest sponsors in our sample aired an average of 496 times each, whereas creatives sponsored by other sponsors aired an average of 517 times each (this difference was not statistically significant; *t* test *p* = 0.8070). This means that consumers were no more exposed to content we excluded. Next, we included all ads sponsored by the less common entities (those ranked as the fourth most prevalent or less in the total data set; *N* = 560 creatives), which included less frequent private sponsors and state-based marketplaces. After accounting for these sampling decisions, our sample contained 78% of all possible creatives (*N* = 749 total creatives), which encompassed 81% of all available health insurance ad airings (non-Medicare) during this timeframe.

We applied additional exclusion criteria similar to the process implemented by Pando et al. (2022) to focus on ads for health insurance-related products. We eliminated ads that did not offer a health insurance product, ads focused on Medicare products (that were missed in the primary exclusion stage), ads for health insurance products in Mexico (but aired in overlapping US and Mexico markets), ads not aired in 2018 (that were incorrectly included in the data set), and ads where more than half of the ad information was missing because of a technical error.

Considering our primary aim, which was to understand how insurance ads depict populations that could benefit from health insurance access (specifically Medicaid expansions) for individuals younger than 65 years, we also excluded ads that mentioned Medicaid in the context of “dual-eligible” Medicare and Medicaid plans. The target population for these plans is distinct from those who could enroll in the Medicaid program. After applying all exclusion criteria, the final analytic sample included 437 English- and 140 Spanish-language creatives that were aired 306,976 times on local television or national cable in 2018.

### Codebook development and key measures

A subset of the authors used an inductive process to develop a codebook implemented in Qualtrics. The team met regularly to watch ads from the sample, review themes that emerged, and discuss how to operationalize different aspects of ads into variables that related to our research questions. Team members reviewed previous codebooks from studies examining ad content (Barry et al., 2018; Pintor et al., 2020) and then adapted and added variables to reflect our interest in the populations depicted in ads. The team also coded each ad for various elements, including the ad language, ad objective, ad sponsor, and any reference to the Medicaid program (these measures are described below). Lastly, Kantar/CMAG provided information about ad length, ranging from 10 to 120 s, which was used to adjust the analyses, also described below. More detailed descriptions of each measure are found in Appendix A.

Our interest was in capturing content that could signal current or future policy beneficiaries, so we developed an exhaustive set of variables describing people. We created variables for the individual and family structures we thought could or should appear in ads (for a list of variables and how each was defined, please see Appendix 1).

Ad objective distinguishes ads that provide any information to a viewer about how to enroll in a plan—coded as an enrollment objective—and those that instead promote an insurer’s reputation—coded as a branding objective. Some ads may have mostly been about branding but included a short appeal with information about how to enroll in a plan; these were coded as enrollment ads. Ads focused on raising awareness about a health condition or service separate from health insurance (e.g., breast cancer screenings) akin to a public service announcement were coded as branding/public service announcement.

Ad sponsor included the private sector (e.g., private health insurance, integrated insurance, and health delivery systems, or insurance brokers), states, and the federal government. We identified two federally sponsored ads (aired 141 times) that advertised the Children’s Health Insurance Program and the NIHSeniorHealth.gov website separately. Unsurprisingly, there were no federal government ads for Healthcare.gov during this period. We created a public sponsor category that combined federally and state-sponsored ads, which we introduce after Table 1.

We identified ads for Medicaid plans as those that included an explicit or implicit reference to Medicaid, either audibly or visually. Ads may have exclusively referred to a Medicaid plan—what we considered a Medicaid-focused ad—or mentioned a Medicaid plan in addition to other offerings from the ad’s sponsor. For the purpose of this analysis, we combined ads explicitly focusing or briefly mentioning Medicaid into a single sample of ads for Medicaid plans. Our motivation for this collapse was that ads with either a narrow focus or brief mention of Medicaid both provide a viewer with exposure to Medicaid. Coders also identified content that advertised a Medicaid plan but did not explicitly refer to it as such, referred to as “Implicit Medicaid” (a common strategy of Medicaid plans, see, e.g., Tallevi, 2018). Coders were instructed to leverage their health policy expertise and use publicly available materials (e.g., websites) to ascertain this implicit Medicaid content. This led to three possible categories of ads: those that did not reference Medicaid; those did reference Medicaid, either explicitly or implicitly; and, within the latter category, those that explicitly referenced Medicaid.

Three authors double-coded a random sample (18.3%) of English-language ads to assess interrater reliability. A single coder coded all of the Spanish-language ads and a sample of English-language ads. To ensure reliability, coders completed instrument training together and communicated regularly to compare and discuss ad coding. All variables in this study exceeded conventionally accepted levels of interrater reliability (*κ* > 0.65). *κ* for each variable are listed in Appendix 1.

### Data analysis

In accordance with similar studies, we analyzed data at the airings level (vs. the creative level) to consider the number of times the depictions—each ad image—were aired on TV. Each creative was aired a variable number of times over the study period both within and across study markets (i.e., some ads were aired as few as 1 time, others were aired as many as 23,921 times). In essence, estimating the frequency of content featured at the airing level weights depictions by how often they were available to the public, so depictions featured in frequently aired ads have higher volumes than depictions featured in less frequently aired ads. Previous research analyzing advertisement content has used a similar approach (see, e.g., Barry et al., 2018).

As the first step in our analysis, we compare the individuals and family structures depicted in ad airings for Medicaid plans to those for non-Medicaid plans. For Medicaid plans, we include two categories as noted above. Next, we report the differences in depictions by sponsor. In analyses testing for statistical differences by sponsor, we adjusted for ad length because we observed systematic differences in length of ad by sponsor type. Longer ads can include more individuals and family structures as part of their additional content.

Last, to make comparisons between the demographics of Medicaid populations in ads compared to the real world—and thus to analyze how accurately or not the images in ads depicted potential enrollees—we drew from the Medicaid and CHIP Payment and Access Commission (MACPAC) analysis of 2018 National Health Interview Survey data (MACPAC, 2020). In 2018, more children under the age of 18 and individuals of color were enrolled in Medicaid as compared with private insurance plans (see Table 4).

## RESULTS

### Summary of sampling frame

Table 1 is a breakdown of the final analytic sample, looking at the distribution of key variables among all ads: those for non-Medicaid plans, those explicitly mentioning Medicaid, as well as the combined category of those explicitly and implicitly mentioning Medicaid. Across all ads, more content aired in English than in Spanish. Less than 10% of ad airings explicitly mentioning Medicaid were in Spanish, compared with close to 20% of non-Medicaid ads. Most airings (78%) were privately sponsored and among private sponsor types, the largest sponsor type across groups were insurance companies. More ad airings overall had an enrollment objective, although we observed differences in the distribution of ads by objective when comparing non-Medicaid and Medicaid content. Whereas slightly less than two-thirds of all ad airings (61.8%) had an enrollment objective, over 80% of airings mentioning Medicaid plans made an enrollment appeal (87.% and 80.6%, respectively, for explicit references vs. the more inclusive explicit and implicit references).

### Individuals depicted in ads, by reference to Medicaid

Results presented in Table 2 display the frequency of individuals and family structures appearing in ads. Considering the sample overall, most ad airings (71%) included depictions of people of color as a patient or potential plan enrollee and more than half included at least one depiction of an individual adult female or male (55.2% and 51.8%, respectively). Just over a quarter of all ads (25.5%) included children and fewer depicted a presumed family structure of two adults with a child (11.6%). These trends were similar among ad airings not mentioning Medicaid. Among content with any reference to Medicaid plans, fewer ads included an individual of color depicted as a patient: 51.7% of ads explicitly mentioning Medicaid and 64.7% of ads either explicitly or implicitly mentioning Medicaid included an individual of color depicted as a patient or plan enrollee. Fewer ads mentioning Medicaid included depictions of two individuals and a child (3.9% and 3.4% for both groupings, respectively) compared with ads not referencing Medicaid plans. Few ads overall included pregnant individuals (4.4%) and no ad airings explicitly mentioning Medicaid included this depiction. All differences between the non-Medicaid versus Medicaid categories were statistically significant, from *χ*^2^ tests.

### Individuals depicted in ads, by reference to Medicaid and by sponsor type

Table 3 reports the individuals and family structures depicted in the overall sample, as well as ads for non-Medicaid plans and Medicaid plans, stratifying by private and public ad sponsors. Privately sponsored ads for non-Medicaid plans included more depictions of two adults and a child, individual adult females and males, and individuals perceived to be in a couple than ads for Medicaid plans. Considering privately sponsored content for Medicaid plans—either explicitly referenced or explicitly and implicitly—there were more depictions of children and individual adult females with a child. Among publicly sponsored ads, non-Medicaid content included a greater percentage of children depicted (53.3%), as well as individual adult females and males (42.6% and 41.0%, respectively). Many of the individual and family structures only appeared in privately sponsored Medicaid content: there were no depictions of an individual adult male with a child or individuals perceived to be in a couple in any publicly sponsored ad airings for Medicaid plans.

In Table 4, we describe the demographic characteristics for populations enrolled in Medicaid in 2018, as well as those enrolled in a non-Medicaid private health insurance plan, without insurance, or enrolled in Medicare. More than half of the individuals enrolled in Medicaid (50.3%) were ages 0–18 years. Among individuals enrolled in non-Medicaid private plans, the majority (66.3%) were adults ages 19–64 years. This was also true of individuals without insurance: 85.8% were ages 19–64 years. Females represent a larger proportion of Medicaid enrollees (55.7% compared with 44.3% identifying as male) and this trend is true of those enrolled in non-Medicaid private plans too. Looking at available racial data, the proportion of Medicaid enrollees who do not identify as white is greater than that among non-Medicaid private health insurance plan enrollees. Among Medicaid enrollees, close to a third identify as Hispanic (32.1%); just over 10% (13.1%) of individuals in non-Medicaid private plans identify as Hispanic. Close to one-fifth of Medicaid enrollees identify as Black, non-Hispanic; half as many are enrolled in non-Medicaid private plans (10.1% of enrollees in these plans identify as Black).

## DISCUSSION

Our results provide evidence that the individuals depicted in advertisements for Medicaid plans differ from those depicted in advertisements for non-Medicaid plans and depictions in Medicaid ads do not reflect populations proportionate to those currently enrolled in the program. Advertisements for Medicaid plans included a greater proportion of single adult females with children and a smaller proportion of two individuals with a child; the latter could be perceived as individuals who parent together and the former, a single mother. These differences could suggest to viewers, which may include policymakers, that the populations who enroll in a Medicaid plan differ from those who enroll in non-Medicaid plans in ways other than eligibility criteria of financial need or health conditions. These depictions may also deter an eligible individual from enrolling in Medicaid if they perceive the program serves select populations, such as single mothers.

Advertisements for Medicaid plans also depicted populations that differ from those currently enrolled in the program and who are eligible for Medicaid but remain uninsured. As so, ads could contribute to the social construction of a target population for Medicaid that is different from the populations enrolling in or who could benefit from public policy benefits. Although children represent the largest group of individuals enrolled in Medicaid (50.3%) and more than 40% (40.1%) of the uninsured who are eligible for coverage, they were depicted in just over one-fifth of any ads explicitly mentioning Medicaid plans. Pregnant people did not appear in any ads for Medicaid plans, yet Medicaid covered 43% of all births in 2018 (MACPAC, 2020). Individuals perceived as male were more commonly included than those perceived as female, yet a larger proportion of Medicaid enrollees identify as female. If you consider health insurance plans as products, it is curious that, in the case of ads for Medicaid plans, the people who were visualized as users of the product differ from those who use the product. Ads for Medicaid plans did not disproportionately include groups commonly perceived as poor and in need of government resources (e.g., Black mothers; see, e.g., Gilens, 1999); this may be an effort to counter stereotypes and expand ideas of who uses and could benefit from the program. This could be an intentional or unintentional choice by those who create advertising content. Additional research is needed to illuminate the process of developing health insurance advertising, including the goals of insurers in promoting their products and what, if any, influence they believe they have on policymaking.

Given the observed differences in the individuals and family structures depicted in publicly and privately sponsored content, it is worth discussing the role of the private sector in health insurance advertising in 2018. The majority of all ads in our sample were sponsored by private entities, which included insurance companies, and more than 90% of ads for Medicaid plans were privately sponsored. In the absence of federal investment in health insurance advertising, the private sector assumed a larger role in 2018 than in prior years (Barry et al., 2018). The submerged state, or the “…conglomeration of federal social policies that incentivize and subsidize activities engaged in by private actors and individuals” and that “…have shrouded the state’s role, making it largely invisible to most ordinary citizens, even beneficiaries of existing policies,” is relevant to consider in light of the private sector filling a void left by reduced federal spending (Mettler, 2010; p.804). Advertisements for Medicaid-managed care plans comprise the bulk of privately-sponsored content for Medicaid plans in our sample and these offer one example of the submerged state. State Medicaid programs contract with private insurance companies to deliver health benefits to Medicaid enrollees based on a per-member, per-month payment (Centers for Medicare & Medicaid Services, 2020). State programs incentivize private insurers to offer a federal social policy benefit and many enrollees are unaware they are receiving a publicly subsidized benefit, limiting their own ability to recognize the personal relevance of public policy (Tallevi, 2018). This has implications for the perceptions current program beneficiaries have of who could or should benefit from health care policies focused on expanding access to Medicaid and, as a result, beneficiaries may be less inclined to add their voices to debates about key health insurance expansions that may benefit them or people within their communities (Michener, 2018).

## LIMITATIONS

These findings should be considered with key limitations in mind. First, we included a broad variable capturing individuals of color in ads; as so, we are unable to make specific inferences about the racial and ethnic characteristics of individuals depicted in Medicaid ads. This limits our ability to add to the evidence of how media disproportionately and systematically depict individuals who identify with a specific racial or ethnic group (e.g., Black women) in public policy or as welfare beneficiaries. Second, there are limitations with our sample: these data include ad airings on broadcast television and national cable, but do not include ad airings on local cable. Our focus was also on television advertisements and we did not analyze the content of health insurance ads airing on other platforms, such as digital ads displayed on social media, radio spots, or those featured in print or on billboards, but these depictions may also contribute to social constructions of insurance beneficiaries. We also did not code all the ads from the three largest private insurance companies in our sample and instead coded what we believe was a representative sample of ads from these insurers. This limits our ability to speak to all depictions in privately sponsored TV ads that viewers may have been exposed to. Further, this study does not assess the impacts to consumers of exposure to the content in advertising. We cannot and do not claim that exposure to specific populations in ads definitively shapes ideas individuals hold of the populations who benefit or could benefit from the Medicaid program. People are exposed to a variety of information that may shape their beliefs and values about who deserves access to health care; we suggest that health insurance advertising is one of many influences on these beliefs and values. There is a need for additional research to explore whether and how health insurance advertising and other forms of media affect attitudes and beliefs about health insurance and if individuals’ ideas of who should receive public policy benefits differs as a result of exposure to content.

## CONCLUSIONS

The Medicaid program is the single largest source of health coverage in the United States (Centers for Medicare & Medicaid Services, 2021a) and its role as a source of health coverage for those with fewer resources may only grow. At the time of this writing, individuals in the United States are encumbered with the coronavirus 2019 pandemic and the resulting economic shocks have left many without employment and employer-sponsored health insurance. In the Spring of 2021, the Biden administration allocated significant resources for health insurance outreach and enrollment support (The White House, 2022). Medicaid, which was a focus of the administration’s efforts, may be one option for those in need of health insurance, and increasing public knowledge of and support for the program is critical to advance lasting federal- and state-level action that will expand program eligibility and allow more to benefit (Park et al., 2021).

Television advertisements for Medicaid plans offer one way to inform public opinion on the need for policy change to expand access to Medicaid. Our results provide evidence that the populations depicted in ads for Medicaid plans do not reflect the populations currently enrolled or who may be eligible for Medicaid benefits. Additional research is needed to explore the content of recent health insurance advertising, especially given the Biden administration’s focus on increasing health insurance outreach and enrollment (Keith, 2021), and to understand the populations depicted, the messages conveyed, and the opportunity for ad content to bolster public support for policy reform.

## Figures and Tables

**TABLE 1:** Characteristics of advertisement airings, by Medicaid focus

	All ads (*N* = 306,976 | % of sample)	Non-Medicaid ads (*N* = 253,095 | 82.5% of sample)	Explicit mentions (*N* = 39,814 | 13.0% of sample)	Explicit and implicit: Medicaid mentions (*N* = 53,881 | 17.6% of sample)
Language spoken				
English-Spoken Ads	256,538 (83.6)	208,843 (82.5)	36,198 (90.9)	47,695 (88.5)
Spanish-Spoken Ads	50,438 (16.4)	44,252 (17.5)	3616 (9.1)	6186 (11.5)
Sponsor type				
Federal sponsor	141 (0.1)	2 (0.0)	139 (0.4)	139 (0.3)
State sponsor	67,435 (21.9)	62,998 (24.9)	3710 (9.3)	4437 (8.2)
Private sponsor	239,400 (78.0)	190,095 (75.1)	35,965 (90.3)	49,305 (91.5)
Private sponsor type				
Insurance company	204,676 (85.5)	157,122 (82.7)	34,366 (95.6)	47,554 (96.5)
Insurance company and health system	31,497 (13.2)	31,119 (16.4)	378 (1.0)	378 (0.8)
Insurance broker/Insurance agency	3227 (1.3)	1854 (1.0)	1221 (3.4)	1373 (2.8)
Objective				
Enrollment	189,695 (61.8)	146,284 (57.8)	34,685 (87.1)	43,411 (80.6)
Branding	103,581 (33.7)	93,111 (36.8)	5129 (12.9)	10,470 (19.4)
Branding/Public service announcement	13,700 (4.5)	13,700 (5.4)	0 (0.0)	0 (0.0)
Ad length				
<30 s	29,429 (9.6)	24,198 (9.6)	3613 (9.1)	5231 (9.7)
30–60 s	277,489 (90.4)	228,839 (90.4)	36,201 (90.9)	48,650 (90.3)
90–120 s	58 (0.02)	58 (0.02)	0 (0.0)	0 (0.0)

**TABLE 2:** People depicted in health insurance advertisement airings, by Medicaid references in ads

	Overall sample (*N* = 306,976)	Non-Medicaid ads (253,095)	Explicit Medicaid (39,814)^b^	Explicit and implicit Medicaid (53,881)^c^
Variable				
Non-White individual depicted as a patient^a^	141,349 (71.0)	117,413 (72.4)	12,277 (51.7)	23,936 (64.7)
Adult female	169,485 (55.2)	151,591 (59.9)	14,225 (35.7)	17,894 (33.2)
Adult male	159,016 (51.8)	140,754 (55.6)	15,833 (39.8)	18,262 (33.9)
Child/children	78,145 (25.5)	64,241 (25.4)	8,168 (20.5)	13,904 (25.8)
Two adults and a child	35,666 (11.6)	33,863 (13.4)	1559 (3.9)	1803 (3.4)
Adult female and child	63,175 (20.6)	43,552 (17.2)	12,168 (30.6)	19,623 (36.4)
Adult male and child	29,028 (9.5)	27,599 (10.9)	1328 (3.3)	1429 (2.7)
Two individuals perceived to be in a relationship	54,656 (17.8)	52,796 (20.9)	1370 (3.4)	1860 (3.5)
Two individuals perceived to be in a nonheterosexual relationship^a^	953 (1.7)	48 (0.1)	905 (66.1)	905 (48.7)
Pregnant individual	13,361 (4.4)	10,047 (4.0)	0	3314 (6.2)
Older adult	66,138 (21.6)	60,073 (23.7)	3309 (8.3)	6065 (11.3)
Injured or disabled individual	5163 (1.7)	5158 (2.0)	0	5 (0.01)

*Note*: Percentages do not add up to 100, because there is overlap of populations depicted in ads and not all ads include people.

a These variables were asked only of relevant ads and reflect a smaller denominator (e.g., the LGBTQ couple variable was asked only of ads with a couple depicted.)

b All *p* testing the difference between ads explicitly focusing on Medicaid plans compared with ads for non-Medicaid plans were statistically significant (*p* < 0.05).

c All *p* testing the difference between ads explicitly or implicitly focusing on Medicaid plans compared with ads for non-Medicaid plans were statistically significant (*p* < 0.05).

**TABLE 3:** People depicted in health insurance advertisement airings, by reference to Medicaid and sponsor type

			Private sponsor (*N* = 239,400)			Public sponsor (*N* = 67,576)		
	Private sponsor (*N* = 239,400)	Public sponsor (*N* = 67,576)	Non- Medicaid (*N* = 190,095)	Explicit Medicaid (*N* = 35,965)	Explicit and implicit Medicaid (*n* = 49,305)	Non-Medicaid (*N* = 63,000)	Explicit Medicaid (*N* = 3849)	Explicit and implicit (*N* = 4576)
Variable								
Non-White individual depicted as a patient^a^	120,727 (68.1)	20,622 (94.7)	98,254 (69.3)	11,339 (49.7)	22,473 (63.2)	19,159 (94.4)	938 (100.0)	1463 (100.0)
Adult female	142,394 (59.5)	27,091 (40.1)	124,737 (65.6)	14,124 (39.3)	17,657 (35.8)	26,854 (42.6)	101 (2.6)	237 (5.2)
Adult male	132,835 (55.5)	26,181 (38.7)	114,916 (60.5)	15,796 (43.9)	17,919 (36.3)	25,838 (41.0)	37 (1.0)	343 (7.5)
Child/children	43,374 (18.1)	34,771 (51.5)	30,673 (16.1)	7239 (20.1)	12,701 (25.8)	33,568 (53.3)	929 (24.1)	1203 (26.3)
Two adults and a child	31,099 (13.0)	4567 (6.8)	29,421 (15.5)	1559 (4.3)	1678 (3.4)	4442 (7.1)	0 (0.0)	125 (2.7)
Adult female and child	59,008 (24.7)	4167 (6.2)	40,230 (21.2)	11,323 (31.5)	18,778 (38.1)	3322 (5.3)	845 (22.0)	845 (18.5)
Adult male and child	28,558 (11.9)	470 (0.7)	27,129 (14.3)	1328 (3.7)	1429 (2.9)	470 (0.8)	0 (0.0)	0 (0.0)
Two individuals perceived to be in a relationship	49,927 (20.9)	4729 (7.0)	48,067 (25.3)	1370 (3.8)	1860 (3.8)	4729 (7.5)	0 (0.0)	0 (0.0)
Two individuals perceived to be in a nonheterosexual relationship^a^	909 (1.8)	44 (0.9)	4 (0.01)	905 (66.1)	905 (48.7)	44 (0.9)	0 (0.0)	0 (0.0)
Pregnant individual	9765 (4.1)	3596 (5.3)	6456 (3.4)	0 (0.0)	3309 (6.7)	3591 (5.7)	0 (0.0)	5 (0.1)
Older adult	64,977 (27.1)	1161 (1.7)	58,936 (31.0)	3309 (9.2)	6041 (12.3)	1137 (1.8)	0 (0.0)	24 (0.5)
Injured or disabled individual	4768 (2.0)	395 (0.6)	4768 (2.5)	0 (0.0)	0 (0.0)	390 (0.6)	0 (0.0)	5 (0.1)

*Note*: Statistical tests between Medicaid-related ads were calculated using logistic regression with non-Medicaid ads as the reference group, controlling for the length of an ad. All *p* for tests of difference in characteristics by Medicaid versus non-Medicaid were statistically significant (*p* < 0.05).

a These variables were asked only of relevant ads and reflect a smaller denominator (e.g., the LGBTQ couple variable was asked only of ads with a couple depicted).

**TABLE 4:** Characteristics of noninstitutionalized US individuals by health coverage, 2018

	Total	Medicaid or CHIP (% of total)	Private (% of total)	Uninsured (% of total)	Medicare (% of total)
Age (years)					
0–18	24	50.3	21.2	13.3	0.7
19–64	60	42.4	66.3	85.8	13.8
65+	16	7.3	12.5	0.9	85.5
Gender					
Male	48.9	44.3	49	55.1	45.6
Female	51.1	55.7	51	44.9	54.4
Race					
Hispanic	18.4	32.1	13.1	37.1	9.3
White, non-Hispanic	61.3	38.9	68.7	41.9	75
Black, non-Hispanic	12.6	21.2	10.1	14.1	10.3
Other non-White, non-Hispanic	7.7	7.8	8.1	7	5.4

*Note*: MACPAC, analysis of NHIS data.

Abbreviations: MACPAC, Medicaid and CHIP Payment Access Commission; NHIS, National Health Interview Survey.

## Key points

- Individuals and family structures depicted in television ads for Medicaid health insurance plans differed from those depicted in ads for non-Medicaid insurance plans.
- Further, television ads for Medicaid plans airing in 2018 did not depict the breadth of populations that may be eligible for the Medicaid program.
- Medicaid pays for slightly less than half of all births in the United States and yet pregnant people were not depicted in any ads for Medicaid plans.

## APPENDIX

**TABLE A1:** Intercoder reliability values and variable definitions

Variable	Kappa	Definition
Language spoken		
English	0.00	Content is primarily delivered in English.
Spanish	0.00	Content is primarily delivered in Spanish.
Sponsor type		
Federal	0.84	Ad is either Medicare or CHIP.
State	0.84	Ads focus on enrollment in individual health insurance plans from a State based Marketplace or traditional Medicaid or CHIP. There may be also be mention of small business insurance run by the state.
Private	0.84	Any ad for an insurance company (can be for-profit or non-profit), insurance agency, broker, health care system, or managed care organization.
Private ad sponsor type		
Insurance company	0.75	Any seller of an insurance plan including for-profit companies and nonprofit organizations.
Insurance company and health system	0.75	Includes organizations like Kaiser Permanente, UPMC, etc. that offer insurance and operate health care facilities.
Insurance broker	0.75	Includes ads for a service that will connect a consumer to insurance. The company does not actually offer insurance. These may be individual brokers or agents, or they may be a company that matches consumers to a variety of plans from other companies.
Ad objective		
Enrollment	0.70	Ads explicitly mention, audibly or visually, enrolling in a health insurance plan and may provide details about the plan(s).
Branding	0.70	Ads provide the insurer name and potentially their market (e.g., Blue Cross Blue Shield of Illinois) but do not provide any appeal to enroll or any explicit information about plans.
Branding/Public service announcement	0.70	Ads provide information to the viewer about issues or resources broader than the health plan. Examples include information presented in celebration of or to raise awareness about a particular cause (e.g., heart health). Message of a Branding/PSA ad will primarily focus on an issue or resource rather than on the insurer or the plan(s) they offer.
People depictions		
Child/children	0.80	The ad includes an individual 18 years old or younger.
Two individuals and a child	0.68	The ad includes two individuals with a child younger than 18, leading the viewer to believe they are parents. This includes LGTBQ parents (prompt to select yes if so). This includes grandparents (prompt to select yes if so).
Individual adult female and child/children	0.73	The ad depicts a single adult female together with a child or children. Ads including an adult female and a child in separate parts of the ad would not be coded here unless they are also depicted in the same scene. If the same child is depicted earlier or later alone, but is also shown in the company of the parent (or vice versa) only count the paired structure – do not also double-count the child as a child alone and the parent alone.
Individual adult male and child/children.	0.64	The ad depicts a single adult male together with a child or children. Ads including an adult male and a child in separate parts of the ad would not be coded here, unless they are also depicted in the same scene. If the same child is depicted earlier or later alone, but is also shown in the company of the parent (or vice versa) only count the paired structure – do not also double-count the child as a child alone and the parent alone.
Adult female	0.74	The ad depicts an adult female, understood to be between the ages of 18 and 65.
Adult male	0.83	The ad depicts an adult male, understood to be between the ages of 18 and 65.
Two individuals perceived to be in a relationship	0.73	The ad includes people perceived to be in a romantic relationship. An example would two individuals walking together holding hands. A couple with a child would not be coded here, but as two parents and a child. This code includes LGTBQ couples.
Pregnant individuals	0.90	The ad depicts an individual or group of individuals who are visibly pregnant.
Older adults	0.84	The ad depicts individuals understood to be over the age of 65 or mentioned as seniors or older adults.
Injured or disabled individual	0.65	The ad depicts an individual with an injury or disability. Example include individuals on crutches or in a wheel chair.
Non-White individual depicted as a patient	0.78	A non-White individual depicted as a patient, including pictured with a health care provider or in a health care setting.
